# Role of the Transforming-Growth-Factor-β1 Gene in Late-Onset Alzheimer’s Disease: Implications for the Treatment

**DOI:** 10.2174/1389202911314020007

**Published:** 2013-04

**Authors:** Paolo Bosco, Raffaele Ferri, Maria Grazia Salluzzo, Sabrina Castellano, Maria Signorelli, Ferdinando Nicoletti, Santo Di Nuovo, Filippo Drago, Filippo Caraci

**Affiliations:** 1 IRCCS Associazione Oasi Maria S.S. – Institute for Research on Mental Retardation and Brain Aging, 94018 Troina, Enna, Italy; 2 Department of Educational Sciences, University of Catania, Catania, Italy; 3 Department of Clinical and Molecular Biomedicine, Section of Psychiatry, University of Catania, Catania, Italy; 4 Department of Human Physiology and Pharmacology, University of Rome “Sapienza”, Rome, Italy; 5 INM Neuromed, Pozzilli, Italy; 6 Department of Clinical and Molecular Biomedicine, Section of Pharmacology and Biochemistry, University of Catania, Catania, Italy; # International PhD Program in Neuropharmacology, University of Catania

**Keywords:** Alzheimer’s disease, Depression, Drugs, Genetic polymorphism, Risk factor, Transforming-growth-factor-β1.

## Abstract

Late-onset Alzheimer's disease (LOAD) is the most common form of dementia in the elderly. LOAD has a complex and largely unknown etiology with strong genetic determinants. Genetics of LOAD is known to involve several genetic risk factors among which the Apolipoprotein E (APOE) gene seems to be the major recognized genetic determinant. Recent efforts have been made to identify other genetic factors involved in the pathophysiology of LOAD such as genes associated with a deficit of neurotrophic factors in the AD brain. Genetic variations of neurotrophic factors, such as brain-derived neurotrophic factor (BDNF), and transforming-growth-factor-β1 (TGF-β1) are known to increase the risk to develop LOAD and have also been related to depression susceptibility in LOAD. Transforming-Growth-Factor-β1 (TGF-β1) is a neurotrophic factor that exerts neuroprotective effects against ß-amyloid-induced neurodegeneration. Recent evidence suggests that a specific impairment in the signaling of TGF-β is an early event in the pathogenesis of AD. TGF-β1 protein levels are predominantly under genetic control, and the TGF-β1 gene, located on chromosome 19q13.1–3, con-tains several single nucleotide polymorphisms (SNPs) upstream and in the transcript region, such as the SNP at codon +10 (T/C) and +25 (G/C), which is known to influence the level of expression of TGF-β1. In the present review, we summarize the current literature on genetic risk factors for LOAD, focusing on the role of the TGF-β1 gene, finally discussing the possible implications of these genetic studies for the selection of patients eligible for neuroprotective strategies in AD.

## INTRODUCTION

Alzheimer's disease (AD) is a neurodegenerative disorder characterized by the presence of β-amyloid in the senile plaques, intracellular aggregates of tau protein in the neurofibrillary tangles, and progressive neuronal loss [1]. AD is mainly characterized by memory loss, with disoriented behaviour and impairments in language, comprehension, and spatial skills able to interfere with the quality of life and normal daily activities. Neuropsychiatric symptoms, such as depression, psychosis and agitation are also frequent in people with AD, and are a common precipitant of institutional care [2].

AD is the most frequent form of dementia in the population under 65 years of age. In AD, genetic variability has an incidence of about 70% [3]. Three decades of genetic research in AD have led to the identification of rare, disease-causing mutations in genes encoding for the β-Amyloid Precursor Protein (APP), presenilin 1 (PSEN1), and presenilin 2 (PSEN2) [4-6]. These mutations cause the majority of early-onset familial AD (EOAD) through an increased production of β-amyloid (Aβ), the accumulation of which is widely thought to trigger both synaptic dysfunction and neurodegenerative phenomena in the AD brain [1, 7]. However, more than 90% of AD cases are of the late-onset form (LOAD), which typically manifests in people older than 65 years and seem to have a separate and largely undescribed genetic etiology.

Genetics of LOAD is known to involve several genetic risk factors among which the Apolipoprotein E (APOE) gene seems to be the major recognized genetic determinant [8-11]. Different studies have demonstrated the essential role of the APOE gene (with the APOE4 allele increasing risk and the APOE2allele decreasing risk) both in familial late-onset and sporadic AD patients [12-15]. Different efforts have been made to identify others genetic factors involved in the pathophysiology of LOAD [16]. Association studies for specific candidate genes, selected because of their known biological function relevant to AD, have been performed for over 350 single nucleotide polymorphisms (SNP) of different genes including interleukin 1A (IL-1A), interleukin 1B (IL-1B), interleukin 1 receptor antagonist (IL-1RN) [17, 18], and methylentetrahydrofolate reductase (MTHFR) [19, 20]. Unfortunately data obtained in association studies have not been always replicated in different samples for different reasons such as the reduced sample size and the single-stage study design which have generally been too small for the moderate effect sizes and the substantial locus heterogeneity that we now know underlies LOAD. Replication studies that utilize a large design population are needed to confirm the association between the identified SNP and the risk to develop LOAD.

Genome-wide association studies (GWAS) represent a new and successful approach to find new candidate genes in AD [21]. We have recently participated in systematic, high-throughput genomic approaches which identified new genetic determinants involved in the pathophysiology of LOAD such as clusterin (*CLU)* and complement component receptor 1 (*CR1)* [22, 23]. These recent studies confirm the central role of genes associated with a defect in peripheral β-amyloid (Aβ) peptide clearance, suggesting that the amyloid cascade hypothesis could be relevant not only in the AD monogenic forms (EOAD), but also in the common and late-onset forms of the disease (LOAD).

According to the “amyloid cascade hypothesis”, oligomeric species composed of aggregated ß-amyloid (Aβ) are believed to cause synaptic dysfunction and finally neurodegeneration in the AD brain [1]. Recent studies suggest that genetic deficits of neurotrophic factors, such as brain-derived neurotrophic factor (BDNF), and transforming-growth-factor-β1 (TGF-β1) might also contribute to increase the vulnerability of AD brain to the neurotoxic activity of Aβ [24-26].

In the present review, we summarize the current literature on the role of TGF-β1 gene variants as risk factors for LOAD, finally discussing the possible implications of these genetic studies for the treatment of LOAD.

## HUMAN TGF-β1: BIOLOGY AND GENETICS

Transforming-growth-factor-β1 (TGF-β1) is a member of TGF-β superfamily, which includes several groups of highly conserved multifunctional cell-cell signaling proteins of key importance in the control of cell growth, differentiation, and embryogenesis, as well as immune suppression and neuroprotection [27-30]. Within the mammalian TGF-β superfamily, TGF-β1, 2 and 3 are important modulators of cell survival and apoptosis [31]. All three TGFβs are synthesized as homodimeric proproteins (pro-TGFβ) and derived from three unlinked genetic loci, TGFB1, TGFB2 and TGFB3, which encode three protein isoforms, TGF-β1, TGF-β2 and TGF-β3 with great structural and functional similarities [32]. TGF-β1 is the most abundant isoform and is highly conserved in primary sequence through evolution [33]. Nucleotide sequences as well as related aminoacid sequences of human, mouse, pork, cow, ape and chicken demonstrate that mature polypeptide of TGF-β1 is conserved 100% across these species with the exception of a single amino acid substitution in the murine peptide. The human TGF-β1 gene, located on chromosome 19q13, contains seven exons of which part of exon 5, exon 6, and exon 7 encode for a precursor protein of 390 amino acids (pro-TGFβ) which is then processed proteolytically to generate the active mature protein of 112 amino acids, a 25 kDa disulphide linked homodimer or heterodimer protein with a broad range of biological functions [34].

TGF-β1 interacts with a high-affinity transmembrane receptor complex consisting of the activin-like kinase 5 (ALK5)/TGF-β type I receptor and the TGF-β type II receptor (TβRII) subunits with the latter having a serine /threonine kinase domain [27]. When TGF-(1 binds to TβRII it induces the assembly of type I and type II receptors into a complex with the subsequent transphosphorylation type I receptor by the type II receptor kinase. The consequently activated type I receptor phosphorylates selected Smads, and these receptor-activated Smads (R-Smads: Smad 2, Smad 3, Smad 5 and Smad 8) then form a complex with Smad 4. Activated Smad complexes translocate into the nucleus, where they regulate the expression of different target genes involved cell proliferation, differentiation, immune suppression and repair after injury [27]. Besides Smad-mediated gene transcription, TGF-β1 activates Smad-independent pathways, including the extracellular-regulated kinase (ERK) pathway [35], the nuclear factor κB (NF-κB) pathway [36], and the phosphatidylinositol-3-kinase (PI3K)/Akt pathway [37, 38]. TGF-β/Smad-indipendent pathways have a key role in mediating different biological effects of TGF-β1 such as cell cycle inhibition, immune suppression and neuroprotective effects [35, 38, 39].

TGF-β1 protein levels are predominantly under genetic control and the promoter region of TGF-β1 gene has been characterized by the group of Kim [40, 41]. All positions are defined relative to the first major transcription start site (position +1). The first 840 bases are a non translated region and codon one begins at position 841. Two distinct TGF-β1 promoter regions have been identified both responsive to autoregulation: a first region located between nucleotides -454 to -323 (first promoter) and a second region between nucleotides +1 to +271 of the TGF-β 1 gene [40]. 

Nine polymorphisms have been identified in the TGF-β1 gene: i) three reside in the promoter region, C-988A, G-800A, C-509T; ii) an insertion (C) is found in the 5' UTR at position +72; iii) two SNPs are in the signal sequence [codon +10 (T/C) or T869C and codon +25 (G/C) or G915C], one in exon 5 (T263I or C788T), and one in each of introns 4 and 5 (713-8delC and C861-20T), respectively [33, 42-49]. Recently, seven of these have been reported as single-stranded conformational polymorphisms and applied to a multicenter study population [45].

The TGF-β1 gene is implicated in a varied range of diseases such as hypertension [47, 48], myocardial infarction [42], atherosclerosis [33, 50], cancer [51-53], fibrosis in chronic liver diseases [54-57], diabetic nephropathy [58], asthma [59], multiple sclerosis [60], rheumatoid arthritis [61] and osteoporosis [62, 63]. An increased expression of TGF-*β*1 has been observed with age [64]. A protective role has been suggested for this neurotrophic factor in longevity [64, 65] and increased plasma levels of bio-active TGF-*β*1 have been found both in male and female centenarians, as compared to younger control subjects [66, 67].

Recently, a specific dysfunction of the TGF-β1 signaling pathway has been demonstrated in AD patients [30, 68] with a reduced expression of TGF-β type II receptor in neurons in an early phase of the disease. AD patients also showed a reduction in the plasma levels of the active (25 kDa) and inactive (50 kDa) forms of TGF-β1 [69, 70] as well as a reduced secretion of TGF-β1 from circulating peripheral blood mononuclear cells [71].****

The central role of TGF-β1 dysfunction in AD pathophysiology has been validated also with *in vitro* and *in vivo* models of AD, where the deficiency of TGF-β1 signaling is associated to the Aβ pathology and neurofibrillary tangle formation [30, 72]. 

TGF-β1 is known to induce the expression of the APP gene in several different cell culture systems [29, 73-76] and might thus increase Aβ production. On the other hand overall data from the literature seem to suggest that TGF-β1 can promote Aβdeposition in cerebral blood vessels, but reduces Aβaccumulation in the brain parenchyma [26, 72].

Aß promotes neurodegenerative phenomena in the AD brain through different mechanisms such as amplification of NMDA toxicity [77], the loss of the pro-survival Wnt neuronal pathway [78], the re-activation of the cell cycle in differentiated neurons [79] and finally the release of pro-inflammatory cytokines from microglial cells [80]. 

TGF-ß1 is known to exert neuroprotective effects against Aβ toxicity by selectively interfering with different steps of the Aß-induced death cascade [30, 38]. TGF-β1 is a well-known inhibitor of cell proliferation that may contribute to keep postmitotic neurons in a differentiated state by inducing the expression of the CDK inhibitors p21 and p27 [35]. Cell cycle inhibition is one among the mechanisms by which TGF-β1 exerts its neuroprotective effect against Aß toxicity [38], another being the prevention of Aß-induced tau hyperphosphorylation *via* the PI3K-promoted inhibition of the tau-phosphorylating enzyme, glycogen synthase kinase-3β (GSK-3ß) [38]. TGF-β1 can also prevents Aß-induced microglia activation [81]. It has been hypothesized that a reduced TGF-β1 signaling might contribute both to microglial activation and ectopic cell cycle re-activation in neurons, two events that finally promote neurodegeneration in the AD brain [30]. 

According to this scenario the impairment of TGF-β1 signaling might represent an early and relevant event in the pathophysiology of AD. It is therefore important to assess whether TGF-β1 gene variants can contribute to the dysfunction of the TGF-β1 pathway observed in AD brain. 

## TGF-β1 GENE IN AD

The possible association between TGF-β1 gene and AD has been tested in different studies in the last ten years [29, 43, 74, 82-89] (Table **1**). Different SNPs have been examined both in the promoter region (G-800A and C-509T) and in the coding region (at codon +10 (T/C) and +25 (G/C), and exon 5 at codon 263) [29, 43, 74, 82-89]. Some of these studies have also examined the relationship between TGF-β1 gene sequences and circulating levels of TGF-β1.

The TGF-β1 G-800A polymorphism is located in a region endowed with an enhancer-like activity (-1132 to -732), while C-509T polymorphism is located in a negative regulatory region (-731 to -453) previously determined to be associated with decreased transcription of TGF-β1 [41]. Data from the literature show that interindividual changes in circulating levels of TGF-β1 might be caused by variability in C-509T polymorphism [32]. When compared with the C allele at TGF-β1 (-509), the presence of the TGF-β1 (-509) T allele was associated with higher transcriptional activity by luciferase assay [74]. Interestingly the group of Grainger [32] found that almost 10% of the genetic variance in the concentration of active plus acid-activatable latent TGF-β1 [(a+l) TGF-β1] was attributable to C-509T polymorphism with plasma TGF-β1 concentrations approximately twice as high in TT carriers compared with CC homozygotes [32].

The two SNPs at codon +10 (T/C) and +25 (G/C) are within the 29-amino acid signal sequence. Signal sequences regulate posttranslational protein synthesis finally affecting the secretion of this cytokine [34]. In particular it has been demonstrated that the transition at codon 25 of G →C causes an Arg25Pro substitution and reduces the secretion of the TGF-β1 [34, 45]. 

The polymorphism at codon 10, with the transition of T→C causes a Leu10Pro substitution, can also exert a substantial effect on TGF-β1 secretion by altering the hydrophobic [alpha]-helix of the signal sequence, thereby reducing its ability to direct protein transport across the endoplasmic reticulum [34]. Conflicting results have been reported on SNPs at codon +10 (T/C) with a study showing that the T allele of codon 10 is associated with reduced levels of protein in serum and reduced secretion in HeLa cells [29], while a recent study has demonstrated that the +10 CC genotype is associated with reduced serum levels of TGF-β1 in patients with Mild Cognitive Impairment (MCI) converted into AD [88]. These conflicting results might be examined also considering that the authors have not analyzed in these studies the correlation between different TGF-β1 gene variants and the concentration of total, active, and latent TGF-β1 (Pro- TGF-β1). In this regard, it is important to note that the pool of TGF-β1 available for binding to a specific receptor(s) and initiating intracellular signaling is governed not only by the total quantity of TGF-β1 secreted (Pro-TGF-β1), but, most importantly, by the amount of TGF-β1 that is activated. Specific ELISA assays should be used in future studies to determine serum levels of total, active, and latent TGF-β1 (Pro- TGF-β1) in AD patients and to establish how the SNP at codon +10 (T/C) can interact with the SNP at codon +25 (G/C) in influencing TGF-β1 secretion.

The association between TGF-β1 gene polymorphisms and AD risk has been widely reported, but results were somewhat controversial (Table **1**). Some groups [83] studied the association between polymorphisms in the IL-6 (-174G>C) and TGF-β1 gene, (-800, -509, +10, +25 and +263) and the risk of dementia [83]. Haplotype alleles of TGF-β1 gene were coded as haplotype numbers 1 through 4 in order of decreasing frequency in the population coding from G-800A, C-509T, +10, +25 and C263T, haplotype 1 = G-C-T-G-C, haplotype 2 = G-T-C-G-C, haplotype 3 = A-C-T-G-C and haplotype 4 = G-C-C-C-C. No association was found in this study between any of the four TGF-β1 gene haplotypes examined and risk of dementia or AD. Unfortunately in this study the authors did not examine the relationship between TGF-β1 haplotypes and TGF-β1 plasma levels in AD patients.

The group of Araria-Goumidi [43] explored the influence of G-800A, C-509T TGF-β1 promoter polymorphisms, together with the +25 polymorphism, on the risk of occurrence of AD in a large case-control population of 678 sporadic AD patients and 667 controls. The -800 and +25 polymorphisms are known to be in strong linkage disequilibrium with -509. This study did not find an association between all three polymorphisms of the TGF-β1 gene and the risk to develop AD, although the authors did not examine the +10 polymorphism which can influence plasma levels of TGF-β1.

The -800 and the -509 polymorphisms have been analyzed in other three case-control studies [74, 82, 85]. In a large case control study (412 AD vs. 406 controls) the authors did not find a significant association between TGF-β1 G-800A, C-509T, T869C polymorphisms and AD, as well as between these gene variants and serum TGF-β1 levels [85]. Differently from previous studies [69-71], these authors did not observe a significant reduction of serum TGF-β1 levels in AD patients, although ELISA was performed only in a small sample of AD patients. On the other hand, other groups [74] found a significantly higher frequency of the -509/TT genotype in AD patients compared to controls, in a large case-control study including 428 late-onset (≥60 years) AD patients and 421 healthy age- and sex-matched controls. The T allele, specifically the -509/TT genotype, was associated with a modest risk for developing AD, whereas genotype or allele frequency distributions for the -800 or codon 263 polymorphisms did not differ between cases and controls. 

The group of Dickson [82] investigated the association of the -509 SNP and AD in a study population which included: i) a group of 203 families with at least two AD affected siblings with a mean age of onset 71.0, ii) a population of 126 African-American (AA) AD cases versus 93 age-matched controls. Results of family-based association analyses for the -509 polymorphism of TGF-β1 show a significant association between the T allele and the risk to develop AD, whereas associations for the TGF-β1 +869 and -509 SNP with AD were not significant in the AA case-control study. Furthermore, the authors found that the -509 SNP could act as an effect modifier of *APOE4* risk by increasing the risk of developing AD in subjects having at least one *APOE4* allele.

Overall, data available in the literature on the T869C polymorphism do not provide evidence of an association between this polymorphism of the TGF-β1 gene and risk to develop LOAD, whereas further studies are needed to confirm the role of C-509T SNP in LOAD (Table **1**).

On the other hand, different studies have been conducted in the last five years to examine the role of the +10 polymorphism as a genetic risk factor for LOAD.

A first study [86], the Honolulu-Asia Aging Study (HAAS), examined the association between the TGF-β1 codon 10 polymorphism and the risk to both AD and vascular dementia (VaD). Allele frequencies were similar in 162 AD and 99 VaD patients compared to 491 healthy subjects, but the frequency of the combined TC + CC genotypes was lower in VaD cases (69.7%) compared to the controls (78.6%) and these two genotypes were associated with a reduced risk for VaD. The TGF-β1 codon 10 polymorphism did not influence the risk to develop AD, but the CC genotype was associated with an increased risk of neocortical plaques [86]. The association of the TGF-β1 codon 10 polymorphism with cerebral amyloid angiopathy (CAA) has been investigated in a study in 167 elderly Japanese autopsy cases, including 73 patients with AD [29]. The authors found that the number of the T alleles positively correlated with the severity of CAA in non-AD patients and APOE non-E4 carriers, but not in AD patients or APOE E4 carriers.

As discussed above, evidence in animal models suggests that TGF-β1 can promote Aβdeposition in cerebral blood vessels, whereas it reduces Aβaccumulation in the brain parenchyma. Data obtained in AD patients seem to be in accordance to preclinical data suggesting that the TT genotype, by increasing TGF-β1 levels, can increase the severity of cerebral amyloid angiopathy, whereas the CC genotype, by reducing TGF-β1 levels, might promote the formation of neocortical plaques. Other groups observed no statistically significant differences in the allele, genotype and haplotype distribution of codon 10 and codon 25 polymorphisms [84, 87], but, because of the very small size of their samples of AD patients (50 and 19 patients, respectively), it is difficult to drawn final conclusions from these studies.

## TGF-β1 GENE IN MCI TO AD

A recent study investigated the role of TGF-β1 codon 10 polymorphism as a genetic risk factor both for MCI and AD in 198 healthy controls (HC), 193 patients with LOAD and 48 patients with MCI [88]. Interestingly the authors found that the CC genotype of TGF-β1 gene significantly increased the risk to develop LOAD, independently of the APOE4 status. On the other hand the genotype and allele frequencies of +25 C → G SNP were similarly distributed in AD subjects, MCI subjects and controls. Interestingly in MCI converted to AD, after a 4-year follow-up, the percentages of both +10 C allele and CC genotype were higher than in stable MCI. The authors also showed that the CC genotype of TGF- β1 gene was associated both with reduced serum level of TGF-β1 and an increased conversion of MCI patients into AD. This was the first study which examines the role of TGF-β1 gene variants in the preclinical phase of AD, demonstrating that an impairment of TGF-β1 signaling can contribute to promote the transition from MCI into AD. Because of the small size of the sample, additional studies are needed to confirm these results in a larger sample, focusing in particular on multiple-domain amnestic MCI patients who are at high risk to develop AD.

We have recently examined TGF-β1 +10 (T/C) and +25 (G/C) SNPs and allele frequencies in a case-control study with 131 sporadic AD patients and in 135 healthy age- and sex-matched controls. We found that allele frequencies of codon +10 polymorphism showed a significant difference between AD patients and controls. Interestingly we observed a different distribution of the +10 (C/C) genotype between LOAD patients and controls, but not between EOAD patients and controls [89]. The homozygous state for the C allele was associated with an increased risk of LOAD (more than twofold) regardless of the APOE4 genotype (Table **1**).

We also examined in this study the influence of the +10 (T/C) and +25 (G/C) polymorphism on the onset of AD-related depression in LOAD patients. TGF-β1 is known to be involved in the pathogenesis of depression [90, 91] and depressive disorders occur in about 30-40% of AD patients influencing the clinical evolution of the disease [92, 93]. We found that LOAD patients with the +10 C/C genotype showed >5-fold risk to develop depression, independently of a history of depression. A significant correlation was also found in LOAD patients between the number of TGF-ß1 +10 C alleles (0, 1, 2) and the severity of depressive symptoms as assessed by the Hamilton Rating Scale for Depression (HAM-D_17_) [89]. 

Depression is known to be a risk factor for the development of AD [94], and the presence of depressive symptoms significantly increases the conversion of MCI into AD [95]. As observed with the +10 (T/C) and +25 (G/C) polymorphism of the TGF-ß1 gene, other genetic variations of neurotrophic factors, such as the brain-derived neurotrophic factor (BDNF) Val66Met functional polymorphism, increase the risk to develop depression in AD patients [25] and also determine a higher risk of disease-progression in patients with MCI [96]. It might be interesting to examine whether the +10 (T/C) functional polymorphism of TGF-β1 acts synergistically with the BDNF Val66Met functional polymorphism in increasing the risk to develop depressive disorders in MCI and/or the following risk of conversion into AD (Table **2**).

## CONCLUSION AND PHARMACOLOGICAL PERSPECTIVES

Recently, the criteria for the clinical diagnosis of AD have been revised by the National Institute on Aging and the Alzheimer’s Association workgroup [97] and new criteria focus on the presymptomatic stage of AD and incorporate biomarkers to identify early stages of AD, susceptible of being treated with disease-modifying drugs [98, 99].

Clinical trials on disease-modifying drugs will be therefore focused, in the next years, on prevention rather than treatment of AD. Neuropsychological tools combined with validated biological markers might be essential to detect the earliest clinical manifestations of AD and might be also particularly useful in monitoring the response to disease-modifying therapies in amnestic MCI patients who are at high risk to develop AD. Presently, there are few biological markers for the early identification of MCI which progresses to AD and MCI which does not progress, among which we should consider the association of elevated tau with low levels of Aβ_42_ in CSF, hippocampal atrophy assessed with magnetic resonance imaging (MRI), and positron-emission tomography (PET) evidence of Aβ deposition [100, 101].

According to this scenario genetic studies might be also important for the identification of new biological markers which can predict the progression from MCI to AD, in particular in amnestic MCI patients which have the highest risk to develop AD. Furthermore these genetic studies should be conducted also considering the presence or the absence of other recognized risk factors for AD such as depression, diabetes, hyperhomocysteinemia [94, 102, 103].

APOE4 allele is the major susceptibility factor for late-onset forms of AD [104], but recent studies, as discussed in this review, suggest that other genetic determinants might also studied to determine specific risk profiles of LOAD patients. 

Neuropsychiatric symptoms may be among the earliest symptoms of preclinical stages of AD and targeting them therapeutically might delay the transition to dementia. Genetic studies of neuropsychiatric symptoms, in particular depression, are therefore important both for the diagnosis and treatment of LOAD. Genetic variations of neurotrophic factors, such as BDNF and TGF- β1, are associated with the risk to develop depression in AD patients and, most importantly, increase the conversion of MCI patients into AD. Longitudinal GWAS in amnestic MCI patients with or without depressive symptoms should be therefore conducted in the next years to examine whether genetic variations of neurotrophins of BDNF and TGF-β1 can affect the rate of conversion into AD by increasing the risk to develop depressive symptoms (Table **2**). However these studies should also consider the contribute of genetic variations in other neurotrophins genes, such as the Nerve Growth Factor (NGF) locus, which can influence the occurrence of LOAD [105].

Genetic studies will be also important for the selection of patients eligible for neuroprotective strategies aimed at rescuing neurotrophin signaling and preventing the progression of AD (Table **2**). 

Rescue of TGF-β1 signaling might represent a new strategy to promote neuroprotection in amnestic MCI patients at high risk to convert into AD [30]. Different psychotropic drugs are known to increase TGF-β1 signaling such as lithium, agonists of group II metabotropic glutamate receptors, and antidepressants [26]. 

Lithium has neuroprotective action in animal models of AD, not only via the inhibition of GSK-3(, but also through other mechanisms, including reduction of Aβ production [106, 107, 91] and the release of TGF-β1 [26]. In patients treated with lithium for psychiatric disorders, the risk of developing AD is reduced [108, 109]. A recent placebo-controlled clinical trial in patients with amnestic MCI showed that long-term lithium treatment slow the progression of cognitive decline and also reduces CSF concentration of P-tau [110], but presently we do not know whether these disease-modifying properties of lithium might be related to TGF-β1 gene variants and/or to a rescue of TGF-β1 signaling.

Agonists of group II metabotropic glutamate receptors protect cortical neurons against ß-amyloid toxicity through the release of TGF-β1 from glial cells [111]. Orthosteric mGlu2/3 receptor agonists are under development for the treatment of schizophrenia [112, 113]. Therefore, these drugs might be helpful for the treatment of psychotic symptoms which are common in the early stage of AD [114]. However, the neuroprotective properties of orthosteric mGlu2/3 receptor agonists might be also evaluated in amnestic MCI patients at high risk to convert into AD.

Finally, different second-generation antidepressant drugs, including venlafaxine, paroxetine and sertraline, significantly increase circulating TGF-β1 levels in patients with major depression [115-117]. It is known that a long-term treatment with antidepressants can reduce the risk to develop AD [118]. On the other hand, studies of antidepressants for depression in AD show conflicting results, with several negative findings reported in recent large trials [119]; therefore, the efficacy of antidepressants as first-line treatment of depression in AD has been recently reconsidered because of the absence of benefit compared with placebo and increased risk of adverse events [120]. Furthermore, depressed MCI patients with a poor response to antidepressants are at an specially increased risk of developing dementia [95]. It will be interesting to examine whether genetic variations of TGF-β1 (i.e. the +10 CC genotype) can influence the rate of LOAD in long-term treated depressed patients and/or the response to antidepressants in MCI or AD patients.

## Figures and Tables

**Table 1.** Overview of the Studies Examining the Association Between TGF-β1 Gene Variants and the Risk to Develop AD

Polymorphism	Association with AD	Refs
	The number of the T alleles positively correlated with the severity of cerebral amyloid angiopathy in non-AD patients	[29]
Codon+10(T/C)	CC genotype significantly increased the risk to develop LOAD, independently of the APOE4 status CC genotype associated both with reduced serum level of TGF-β1 and an increased conversion of MCI patients into AD	[88]
	CC genotype associated both with an increased risk of LOAD regardless of the APOE4 genotype and a >5-fold risk to develop depression in AD	[89]
	CC genotype associated with an increased risk of neocortical plaques	[86]
C-509T	Significant association with AD for T allele with the -509 SNP acting as an effect modifier of APOE4 risk	[82]
C-509T, T869C	For the case-control study in the AA population, there were no associations between the TGF-β1 SNPs and AD	[82]
G-800A, C-509T, codon+25(G/C)	No association between all three polymorphisms and the risk to develop AD	[43]
G-800A, C-509T, codon+263(C/T)	-509 TT genotype associated with a modest risk to develop AD genotype and allele frequency distributions for the -800 or codon +263 polymorphisms did not differ between cases and controls	[74]
G-800A, C-509T, codon+10(T/C), codon+25(G/C), codon+263(C/T)	No association between any of the four TGF-β1 gene haplotypes examined and the risk to develop dementia or AD and no data on TGF-β1 plasma levels	[83]
Codon+10(T/C), codon+25(G/C)	No statistically significant differences in the allele distribution	[84, 87]
G-800A, C-509T, T869C	No association was found in this study	[85]

**Table 2.** Future Research Needs

Confirm the role of SNP at codon +10 (T/C) and C-509T SNP in LOAD through genome-wide association studies (GWAS)
Examine how the SNP at codon +10 (T/C) interact with the SNP at codon +25 (G/C) and C-509T SNP in determining serum levels of total, active, and latent TGFβ1 in AD patients
Validate in a large sample population of amnestic MCI patients the role of the CC genotype of TGF- β1 gene as a risk factor for the conversion of MCI into AD
Study in amnestic MCI patients the possible interaction between the SNP at codon +10 (T/C) and the BDNF Val66Met functional polymorphism in increasing the risk to develop depressive disorders and/or the following risk of conversion into AD
Examine whether genetic variations of BDNF and TGF-β1 can influence the response to antidepressants in MCI or AD patients
Identify at-risk patients with a genetic deficit of TGF-β1 to assess whether drugs able to rescue TGF-β1 signaling (i.e. lithium, agonists of group II metabotropic glutamate receptors and antidepressants) can prevent the progression from MCI into AD
